# Hominin Population Structure, Mating Systems, and Intrasexual Competition: Implications for Craniofacial Robusticity and the Self-Domestication Hypothesis

**DOI:** 10.1007/s12110-025-09498-6

**Published:** 2025-07-25

**Authors:** Grant S. McCall

**Affiliations:** Department of Anthropology, Tulane University and Center for Human-Environmental Research (CHER), 8721 Plum St., New Orleans, LA 70118 USA

**Keywords:** Self-domestication hypothesis, Craniofacial robusticity, Population density, Operational sex ratios, Hunter-gatherers

## Abstract

The self-domestication hypothesis has made significant contributions to our thinking about hominin evolution and ecology. It has struggled, however, to find compelling and testable causes of reductions in hominin aggression and violent behavior. This paper examines variability among hominin mating systems and imbalances in operation sex ratios (OSRs) as a potential factor influencing levels of aggression resulting from male intrasexual competition, i.e., male-male competitive aggression. This paper uses multivariate generalized linear modeling (GLM) to examine data from modern hunter-gatherer societies having to do with the causes and consequences of OSR imbalances (as reflected by levels of polygamy) and male intrasexual competition. This paper focuses especially on the role of population density as a potential source of OSR variability. This paper shows that population density correlates strongly with hunter-gatherer OSRs and that this relationship remains strong when controlling for the effects of other potentially intercorrelated variables. This paper shows that, among modern hunter-gatherers, lower population densities lead to greater imbalances in terms of OSRs and therefore higher levels of male-male aggression. This implies high levels of male intrasexual competition among early hominins given likely conditions of very low population densities. The paper closes by proposing some strategies for investigating hominin demographic patterning prehistorically and predicting OSR imbalances, as well as intrasexual competition, based on that information.

**Supplementary Information:**

The online version contains supplementary material available at 10.1007/s12110-025-09498-6.

## Introduction

Over the last two decades, the “self-domestication” hypothesis has emerged as a potential explanation for a suite of biological and cultural changes experienced by Pleistocene hominins, including reductions in craniofacial robusticity. The self-domestication hypothesis argues that, at some point in the past, hominins underwent a major reduction in the prevalence of aggressive behavior, instead shifting toward social relationships of cooperation and coalitionary behavior (Hare et al., 2012; Gibbons, 2014; Cieri et al., 2014; McCall, 2014; Theofanopoulou et al., 2017; Hare, 2017; Benítez-Burraco & Kempe, 2018; Gleeson & Kushnik, 2018; Thomas & Kirby, 2018; Sanchez-Villagra & van Schaik 2019; Wrangham, 2019; Gleeson, 2020; Benítez-Burraco & Elvira-García, 2023; Gleeson & Wilson, 2023; Summers & Summers, 2023). This shift, in turn, led to anatomical changes, including reductions in robusticity, either as the result of linkages with the neuroendocrine system or other dynamics having to do with altered selective conditions experienced within hominin reproductive systems.

Though the self-domestication hypothesis has garnered significant support, the exact adaptive context at the root of the phenomenon has remained a more open question. For example, two popular explanations include: (1) the development of spoken language, which allowed hominins to verbally conspire in resisting would-be dominators (Progovac & Benítez-Burraco, 2019; Wrangham, 2019, 2021); and (2) the development of effective projectile weapon “killing-at-a-distance” technology, which eliminated the physical advantages of larger and more aggressive would-be dominators (Wrangham, 2019, 2021; see also Sanchez-Villagra & van Schaik 2019). Such ideas (and others like them) are problematic in the sense they rely on the emergence of various features of human uniqueness, which are generally difficult to detect directly on the basis of archaeological or paleontological evidence. This paper offers a new perspective based on modern hunter-gatherer variability focusing on population structure and mating systems and their implications for intrasexual competition among males. Specifically, this paper examines male intrasexual competition as a key structural element influencing both aggression and coalitionary behavior.

The field of evolutionary biology has long embraced the importance of social systems involved in reproduction, including reproductive competition and parental investment (Bateman, 1948; Trivers, 1972; Emlen & Oring, 1977; Clutton-Brock, 1988). The field of evolutionary anthropology, in contrast, has tended to think about hominin mating systems through the lens of either non-human primates (esp. *Pan troglodytes* and *Pan paniscus*) or hypothetical modern human analogs. In approaching human mating systems as an inherently variable phenomenon, this paper utilizes cross-cultural data from modern hunter-gatherers as a means of exploring the factors influencing patterns of intrasexual competition. In doing so, this paper focuses especially on the operational sex ratio (OSR; Emlen & Oring, 1977), which can be defined as the ratio of sexually active males to receptive females. In the context of humans, OSRs are manifested to a great degree in the prevalence of polygamous marriages and differences in the age at first marriage between males and females (Marlowe & Berbesque, 2012). This approach is based on the fact that males with multiple female mates and delays in male age at first marriage effectively skew the ratio of sexually active/available male and female potential mates, providing a major impetus for male intrasexual competition.

This paper finds that, while many variables relate to human hunter-gatherer mating systems, population density is by far the strongest influence. This finding is not unexpected given that population density plays a major role in structuring hunter-gatherer group size, group composition, frequencies of potential mates and intrasexual competitors, and possibilities in terms of group membership flexibility as it relates to the problem of finding mates (Kramer et al., 2017). In short, population density greatly determines how hunter-gatherer societies organize foraging groups, territories, etc., in relation to food resources, and this in turn strongly influences the structural features of individual mating opportunities. Hominin population densities also clearly vary through time in ways that match our expectations from the standpoint of the prevalence of aggressive behavior and intrasexual competition. While our current understanding of Pleistocene hominin demographic patterning does not have the resolution that we might like, it is something that we can examine systematically and for which we can build better inferential methods in the future.

## Background

Skeletal robusticity, and especially craniofacial robusticity, is one of the major distinctive differences between extinct species of hominin and extant modern humans. Indeed, craniofacial morphology—of which robusticity is a key feature—remains a primary basis for the taxonomic distinctions between hominin fossil species within the genus *Homo* (Dunsworth, 2010; Marsh, 2013; Prat, 2022; Tattersall, 2016; Tattersall & Schwartz, 2009; Wood, 2000; Wood & Baker, 2011; Wood & Collard, 1999). Yet, we have generally lacked a compelling explanation for *why* patterns of craniofacial robusticity varied over space and time, as well as between various hominin species. From an adaptive perspective, the anterior dental loading hypothesis (Smith, 1983) posited that craniofacial robusticity resulted from the production of bite force in the utilization of “teeth-as-tools” (see also Willman, 2016). Yet, upon more systematic evaluation, there are biomechanical reasons for doubting this hypothesis (Anton, 1990; O’Connor et al., 2005; Holton, 2009; Weaver, 2014), as well as the fact that there are no major recognizable differences in terms of the tools produced by the various hominins responsible for producing Middle Paleolithic and Middle Stone Age stone tool technologies during the Upper Pleistocene in spite of major differences in terms of patterns of craniofacial robusticity.

In contrast, the self-domestication hypothesis proposes “that late human evolution was dominated by selection for intragroup prosociality over aggression” (Hare, 2017: 157). This behavioral shift led to significant changes in the neuroendocrine system, which were linked with observed reductions in robusticity and other anatomical changes via various genetic/selective mechanisms (Hare et al., 2012; Gibbons, 2014; Cieri et al., 2014; McCall, 2014; Theofanopoulou et al., 2017; Benítez-Burraco & Kempe, 2018; Gleeson & Kushnik, 2018; Thomas & Kirby, 2018; Sanchez-Villagra & van Schaik, 2019; Wrangham, 2019; Gleeson, 2020; Benítez-Burraco & Elvira-García, 2023; Hare & Wilson, 2023; Summers & Summers, 2023). In this model, later hominin populations—particularly early modern humans and their immediate ancestors—experienced selective conditions that favored reductions in their levels of aggressive behavior, which led to changes in both the neuroendocrine system as it relates to aggressiveness and reductions in robusticity.

In discussing the apparent association between behavioral and anatomical features within the self-domestication hypothesis, Wilkins and colleagues (2014) identify a putative mammalian “domestication syndrome” (see also Sánchez-Villagra, 2022). This is, in essence, is a generalization about the tendency of connected package of anatomical changes to occur in association with reductions in aggressiveness through the process of domestication or self-domestication. According to Gleeson and Wilson (2023: 3), although this package of connected features is quite variable, it includes reductions in body size, reductions in brain size, changes in craniofacial morphology, reductions in tooth size, changes in skin/coat pigmentation, changes in the number of vertebrae, and changes in terms of the timing of sexual receptivity and reproductive output (see also Leach, 2003). The domestication syndrome has been observed across numerous human-domesticated species (see Gleeson & Wilson, 2023, for review) and it has also been argued to characterize putatively self-domesticated species, such as bonobos (*Pan paniscus*; Hare et al., 2012) and marmosets monkeys (family Callitrichidae; Ghazanfar et al., 2020). The human self-domestication hypothesis argues that reductions in craniofacial robusticity are an element of such a domestication syndrome.

In this respect, Cieri and colleagues (2014) argue that reduced aggression in hominins was tied to biological changes in the neuroendocrine system having to do with reductions in the production of androgen and testosterone, which are connected with aggressive behavior across a wide range of animal taxa (Wingfield et al., 1990), including humans (Mazur & Booth, 1998). This process reduced facial robusticity through the genetic linkage of that trait with the genes responsible for decreased aggressiveness in what Cieri and colleagues see as the “feminization” craniofacial morphology stemming from lower levels of androgen production. In the same vein, Hare and colleagues (2012) invoke the self-domestication hypothesis in explaining the anatomical differences between bonobos (*Pan paniscus*) and chimpanzees (*Pan troglodytes*), suggesting that the isolation of a chimpanzee ancestor south of the Congo River basin triggered a series of evolutionary processes that resulted in selection for reduced male aggression. Specifically, Hare and colleagues (2012) attribute this selection process to the availability of larger and higher-quality foraging patches and an absence of competition with both other conspecific chimpanzee groups and gorillas (*Gorilla gorilla*). These conditions allowed bonobo females to form stable coalitions with which they could resist domination from aggressive males. As a result, elevated aggressiveness in males began to harm male fitness, whereas male ability to form cooperative alliances with female kin group members and prospective mates increased selective fitness. Hare and colleagues (2012) argue that bonobos therefore exhibit reductions in body and brain size, as well as what the authors call the “juvenilization” of the cranium—an idea overlapping with the putative “feminization” of the skull proposed by Cieri and colleagues (2014) but relying on different neuroendocrine pathways and developmental processes.

In exploring the developmental associations between the neuroendocrine basis of aggressive behavior and the various anatomical features of the domestication syndrome, Wilkins and colleagues (2014) point to neural crest cells (NCCs) as the source of the linkage. NCCs are a crucial group of stem cells in vertebrates that are responsible for both the development of organs related to neuroendocrine system underlying aggressive behavior (especially the adrenal system) and the various anatomical features of the domestication syndrome (Sánchez-Villagra, 2022). Wilkins and colleagues suggest that changes having to do with reductions in aggressiveness were linked with the biological features of the domestication syndrome via the evolutionary developmental role of NCCs in both phenomena.

The NCC model of the domestication syndrome is not without its critics. For example, Sanchez-Villagra & van Schaik (2019) demonstrate the inherent difficulty of directly testing hypotheses having to do with NCCs in hominins—or, for that matter, other mammals. Johnnson and colleagues (2021) emphasize variability in the features of the domestication syndrome across mammalian taxa, as well as the fact that many common features of the domestication syndrome are apparently unrelated to the developmental role of NCCs. They also point out that there is little direct evidence for the pleiotropic association of the behavioral and biological features of domestication via NCCs as might be demonstrated through genomic mapping or similar approaches. Lord and colleagues (2020) go further in suggesting that the putative domestication syndrome is so vaguely defined and multifarious in its manifestation as to undermine the belief in its existence altogether.

More recently, Gleeson and Wilson (2023) defend the existence of the domestication syndrome but argue that it is the result of altered conditions of reproduction and sexual selection induced by captivity or other forms of human influence/proximity. Instead of a universal genetic explanation, such as the NCC model, Gleeson and Wilson argue for a connected suite of biological changes with multiple potential genetic bases and developmental pathways resulting from shared disruptions to patterns of male intrasexual selection and female mate choice. In this view, Gleeson and Wilson effectively eliminate the role of selection for “tameness” in terms of genetic linkages between that behavioral feature and the emergent anatomical characteristics of the organisms in question. This view is noteworthy as this paper also relies on alterations to the conditions of male intrasexual competition and female mate choice as drivers of hominin self-domestication.

### Explaining Potential Reductions in Hominin Aggression

A major question surrounding hominin self-domestication hypothesis is, what caused the reduction in aggressiveness that induced the biological changes leading to reductions in craniofacial robusticity? A few specific scenarios have been offered to explain the reductions in hominin aggressive behavior, such as the origins of verbal language or the development of effective projectile weapon technologies (Murphy, 2019; Progovac & Benítez-Burraco, 2019; Sanchez-Villagra & van Schaik, 2019; Thomas & Kirby, 2018; Wrangham, 2019, 2021; Benitez-Burraco 2020; Benitez-Burraco and Progovac 2020; Gleeson, 2020). Such hypotheses, however, have tended to be ambiguous and problematic for reasons having do with their archaeological/paleontological visibility and their timing. There is considerable debate concerning when verbal language and effective projectile weaponry appeared among hominin populations in different regions. There are also broader epistemological issues in terms of the answerability of such questions based on prehistoric evidence.

In arguing for hominin self-domestication via the emergence of spoken language, for example, Wrangham (2019, 2021) suggests that language allowed hominins to form “verbal conspiracies” to collectively resist dominant males by forming coalitions more effectively. Wrangham (2021) also adds that spoken language would have allowed hominin group members to verbally conspire to more effectively “execute” problematic would-be dominators by forming plans in secret and conducting coordinated sneak attacks. This idea overlaps with the projectile weapon explanation of hominin-self domestication, which suggests that the physical superiority of potential dominant males was supplanted by the ability to kill at a distance with projectiles (Boehm, 1997, 2009; Bowles & Gintis, 2011; Gintis et al., 2015; cf. Wrangham, 2021). Both explanations rely on the emergence of a particular feature of human uniqueness, the timing of which is difficult to identify based on archaeological and/or paleontological evidence.

In terms of the origins of verbal language, there is tremendous disagreement and ambiguity surrounding exactly when the modern human speech capability emerged (Corballis, 2002; Lieberman, 1998, 2007; Schepartz, 1993; Tattersall, 2018). One school of thought considers it likely that verbal language originated sometime after the appearance of the genus *Homo*, perhaps in association with the striking encephalization of Middle Pleistocene hominins (e.g. d’Errico & Vanhaeren, 2009; Stout & Chaminade, 2012). Another school of thought associates the origins of spoken language with origins of anatomically and behaviorally modern humans in the Upper Pleistocene—as late as 50 ka (e.g. Lieberman, 1998, 2007; Corballis, 2002). While potentially significant differences exist between modern humans and early hominins in terms of features such the larynx, mandible, and ear (Lieberman, 2007; Stoessel et al., 2016; Bermejo-Fenoll et al., 2022; Urciuoli et al., 2022; Conde-Valverde et al., 2024), much ambiguity remains (see Albessard-Ball & Balzeau, 2018 for review).

Neanderthals, for example, are a hominin species that lived contemporaneously with early modern humans and that overlapped in their spatial distribution in at least Europe and the Near East. Both hominin species possessed very similar patterns of both stone tool technology and foraging behavior for much of the Upper Pleistocene (Hoffecker, 2018; McCall, 2014; Villa & Soriano, 2010). Neanderthals and modern humans also had nearly identical brain sizes and both produced symbolic objects, such as perforated shell, bone, and ivory beads. To many, these facts and others indicate that both Neanderthals and early modern humans shared the cognitive capacity for producing language (Zilhão et al., 2010; Leder et al., 2021). Yet, the two species of hominin have dramatically different patterns of craniofacial robusticity—which is one of the main sets of anatomical differences distinguishing them, and also one of the main issues at stake in terms of the self-domestication hypothesis.

The issue of timing similarly troubles of the potential role of projectile weaponry in the self-domestication hypothesis. As Wrangham (2021) himself points out, there is little evidence for effective projectile weaponry, such as spear-throwers or bow-and-arrow technology, until fairly late in the Upper Pleistocene. The best claims for early complex projectile weapon systems would appear to belong to the later Middle Stone Age (MSA) of Southern Africa, such as use-wear/impact fractures identified on backed blades in the Howiesons Poort Industry at ~ 65 ka (e.g. Pargeter, 2007; Lombard & Phillipson, 2010). For one thing, this inference of Howiesons Poort bow-and-arrow usage is not a settled issue (see McCall and Thomas 2012 for review). For another thing, even if we accept this inference as valid for the current sake of argument, then Howiesons Poort Industry in Southern Africa is followed by another ~ 30 ka of non-microlithic MSA stone tool technology in which such direct evidence for bow-and-arrow use is lacking.

Finally, there is also evidence for similarity in terms of projectile weapon usage by Neanderthals and early Upper Paleolithic modern humans. For example, Rhodes and Churchill (2009) conclude that Neanderthals and early Upper Paleolithic modern humans were indistinguishable in terms of their respective osteological evidence having to do with throwing behavior. Additionally, based on experimental evidence, Churchill and colleagues (2009) argue that the famous Shanidar 3 Neanderthal rib cage “stab” wound was, in fact, the result of a low-kinetic-energy projectile weapon—in other words, that Shanidar 3 represents an instance in which a Neanderthal was fatally wounded by an (apparently) effective projectile weapon. Churchill and colleagues raise the possibility of the Shanidar 3 wound resulting from violence committed by sympatric early modern humans, although many scholars believe that Shanidar 3 predates the second arrival of Upper Pleistocene early modern human populations (e.g. Trinkaus, 1987). At a minimum, the evidence concerning the patterning and timing of the earliest effective projectile weaponry and its relationship to reductions in robusticity are ambiguous.

Such ambiguity in the timing associated with the hominin self-domestication hypothesis is exemplified by its heavy overlap with the *reverse-dominance hierarchy* model (Boehm, 1993, 1997, 2009). Both ideas focus on the putative mechanisms through which hominins reduced levels of violence and domination by forming increasingly cooperative social relations via coalitionary tactics, and Boehm’s ideas are employed extensively by Wrangham (2019, 2021) in his arguments for hominin self-domestication. For the most part, Boehm is noncommittal about the timing of the emergence of the reverse-dominance hierarchy, however, he does point to Middle Pleistocene developments in terms of both projectile weaponry and the potential emergence of language as factors in terms of the coalitionary resistance of potential dominators. Wrangham (ibid) sees hominin self-domestication as the root of the speciation event that occurred among Middle Pleistocene hominins, such as *Homo heidelbergensis*, with Upper Pleistocene *Homo sapiens* as its descendent. Gintis and colleagues (2015), on the other hand, see the reverse-dominance hierarchy as corresponding with the origin of large-brained members of the genus *Homo*, which occurred perhaps at the beginning of the Pleistocene.

While the overlapping elements of the reverse-dominance and self-domestication hypotheses have been applied to the Lower and Middle Pleistocene, there are major shifts in hominin craniofacial robusticity across the Upper Pleistocene and even into the Holocene. On the one hand, reductions in craniofacial robusticity continue within anatomically modern human populations across the Upper Pleistocene, with early modern human crania, such as those from Omo, Skhul, Qafzeh, and Klasies River, being much more robust than those from more recent periods (Lahr & Wright, 1996). Reductions in certain key aspects of craniofacial robusticity continued and even seem to have accelerated from the terminal Pleistocene through the Holocene (Henneberg, 1988; Lahr & Wright, 1996; Lieberman, 1996; Leach, 2003; Klein, 2009). On the other hand, later Upper Pleistocene modern humans lived contemporaneously and often sympatrically with Neanderthals (and perhaps other archaic species) for thousands of years.

The hominin self-domestication hypothesis may well be germane to explaining Lower and Middle Pleistocene variability in terms of hominin robusticity, including the changes seen in the emergence of anatomically modern humans from more robust hominin ancestors, as Wrangham (2019, 2021) suggests. In my opinion, however, the causal mechanisms involved in the hominin self-domestication hypothesis cannot be accepted unless they are also capable of explaining the changes experienced by late Pleistocene and even Holocene human populations in terms of craniofacial robusticity. In this respect, appeals to mechanisms such as the emergence of spoken language or efficient projectile weaponry seem unlikely candidates in explaining potential reductions in aggression among human populations they relate to the self-domestication hypothesis.

### Hominin Mating Structure, Intrasexual Competition, and Self-Domestication

Another overlapping perspective in explaining putative hominin self-domestication has to do with the issues of mating structure and intrasexual competition (Cieri et al., 2014; Gleeson & Kushnick, 2018; Gleeson & Wilson, 2023; Wrangham, 2019, 2021). This viewpoint prioritizes levels of male aggression related to competition for female mates in terms of the balance between direct violent male-male conflict and female mate choice. In short, at least under certain conditions, higher levels of aggression would be advantageous for males in confronting other competing males and in dominating potential female mates. The idea here is that self-domestication occurred as coalitionary tactics emerged in resisting dominating males (Wrangham, 2019, 2021; see also Boehm, 2009) and female mate choice began to favor less aggressive males with greater inclinations toward cooperation and parental investment (Gleeson & Kushnick, 2018). On the one hand, ideas about intrasexual competition have been related to the previously discussed explanations, such as the development of killing-at-a-distance technology and the origins of spoken language. They have to do with the supplanting of physical strength and aggression with either technological or social strategies for resisting dominating males. On the other hand, intrasexual competition articulates with a large and well-developed body of evolutionary biological theory that is highly relevant to the self-domestication hypothesis.

Since the time of Darwin (1871), much of the field has emphasized the role of polygamous mating structures in fostering intrasexual competition (usually) between males and other males (Clutton-Brock et al., 2006). Early on, Darwin (1871) noted that secondary sexual characteristics, such as weaponry and ornamentation, did not seem likely to help an individual survive in their environment—in fact, often quite the opposite—but were rather related to intrasexual competition. A century later, Trivers (1972) updated Darwin in arguing that, because the potential rate of reproduction (PRR) for males varies inversely with levels of parental investment, male reproductive success is often limited by mating access to females. Therefore, there are potential reproductive limitations imposed by male-male competition, female mate choice, or some combination of both. In this way, with the exception of truly monogamous species (which are rare), intrasexual competition constitutes the key determinate of reproductive success and, concomitantly, evolutionary change.

In their synthesis, Emlen and Oring (1977) argue that the ratio of reproductively active males to receptive females—what they call the operational sex ratio (OSR)—depends on a rather limited set of ecological constraints and has broad consequences for patterns of organism ethology. Emlen and Oring (1977: 216 [emphasis added]) argue: “*The greater the potential for multiple mate monopolization, the greater should be the potential intensity of sexual selection and the tendency for polygamy.*” In this way, Emlen and Oring recognized that external ecological factors influence *polygamy potential*, or the extent to which environmental constraints permit the monopolization of the limiting sex. In this sense, OSR and polygamy potential offer a powerful framework for relating ecological constraints, organism social structure, and patterns of individual behavior.

The concepts of OSR and polygamy potential have not been considered much in syntheses of hominin evolution and even less among modern human hunter-gatherer societies (though see Marlowe & Berbesque, 2012). This is especially unfortunate because, as this paper will show, there is tremendous variability in terms of OSRs and polygamy potential among modern hunter-gatherer populations, which is highly relevant to issues of evolutionary ecology inherent to hominin evolution and the origins of modern humans. Though sometimes regarded as essentially monogamous, modern humans exhibit tremendous variability in terms of their mating systems (Schacht & Kramer, 2019). This variability fundamentally structures patterns of male intrasexual competition and female mate choice and, for that reason, it is surprising that theoretical scenarios having to do with self-domestication have not examined the potential role mating structure variability more closely.

There is strong evidence that imbalances in OSRs can be a major determinant of aggression and violence among small-scale human societies—though the direct observation involved in the calculation of OSRs are generally problematic, as I will discuss further in the next section. For example, Gat (1999) makes the connection between polygyny and gerontocratic cultural systems, since polygyny tends to have the effect of delaying male age at first marriage. In effect, this leads to high frequencies of young unmarried males who are in direct competition with one another for mates (Daly & Wilson, 2022; Wilson & Daly, 1985). This can, in some instances, directly cause violence between unmarried males in the context of intrasexual competition. However, it also frequently leads to violent acts by young males against members of neighboring social groups, and is, in this way, the source of raiding, feuding, abduction, and a wide range of other manifestations of violence. Otterbein (2020), for example, finds that large populational proportions of unmarried males provide an impetus for attacking one’s neighbors (for first-hand ethnographic accounts, see also Warner, 1931; Meggitt, 1962; Oswalt, 1967; Tindale, 1974; Divale & Harris, 1976; Peterson & Taylor, 1998; Ellsworth & Walker, 2014).

It is obvious that polygamy is not the *only* cause of violent behavior among hunter-gatherer societies. However, there is abundant evidence that imbalances in OSRs and levels of violent behavior are directly correlated (Marlowe & Berbesque, 2012). This should be sufficient to demonstrate the fact that variability in the imbalances of OSRs have been a major influence on the levels of violence found among human societies and presumably among our hominin ancestors. Therefore, patterning in terms of polygamy potential among modern hunter-gatherers represents a productive approach for thinking about the issues of aggression and the nature of mating systems related to the hominin self-domestication hypothesis. It also provides an alternative to previous explanations of hominin self-domestication via appeals to various features of human uniqueness, such as spoken language, projectile weaponry, etc.

### OSRs and their Analytical Correlates Among Modern Hunter-Gatherers

There is a basic set of problems inherent to the calculation of OSRs for modern human groups based on ethnographic research: the observations necessary to calculate literal OSR values are nearly impossible to make directly. Both individual sexual activity and/or receptivity are concealed and are outside of the realm of ethnographic data collection for a wide range of both practical and ethical reasons. Despite this problem, there have been several attempts to calculate OSRs for various modern human societies and especially hunter-gatherers based on more easily observed proxies.

Of these attempts, the most sophisticated is that of Marlowe and Berbesque (2012), who calculate OSR as the ratio of reproductively active male-days to reproductively active female-days during a given year. In doing so, they use ages at first marriage as a proxy for the beginning of reproductive activity, which is especially important since there is more variability in the age of males at first marriage than there is for females. In short, males tend to marry later than females and there are some hunter-gatherer societies in which males tend to marry *much later* than females. Marlowe and Berbesque (2012) also note that male ages at first marriage correlate strongly with levels of polygyny.

The calculation of OSRs for human groups is a sensible way of facilitating comparisons between human mating system variability with that found among non-human primates. For the purposes of this paper, however, rather than statistically transforming variables having to do with polygamy and age at first marriage in the calculation OSRs, I believe that it makes more sense to simply utilize use these variables directly in examining the determinates of mating structure among modern hunter-gatherers.

## Materials and Methods

This paper utilizes the dataset provided in Binford’s (2001) synthesis of hunter-gatherer variability. Binford provides data concerning a wide range of socioeconomic and environmental variables for 339 modern and historic hunter-gatherer cases. These data have been shown to be broadly relevant to a wide range of crucial prehistoric phenomena (Johnson, 2014), including various issues related to hominin evolution (McCall, 2014; e.g. Anderson et al., 2023, though see also Venkataraman et al., 2024). Of these 339 total cases, Binford reports data for the percentage of polygamous marriages (%polygamy) for 191 cases and mean difference between the age at first marriage for males and females (MAAMdiff) for 168 cases. Most environmental, economic, and demographic variables are also reported for these cases, including effective temperature (ET), percentage of calories derived from gathering (GATH), and population density (DENSITY). Subsequent analyses also examine male division of labor (MDIVLAB; i.e., the percentage of calories provided by male foraging activities), mean family size (FAMILY), and male parental investment (MALEPI). See Binford (2001) for full definitions and formulas for these variables.

In this analysis, I eliminated 91 cases of coastal hunter-gatherers in which certain demographic and environmental variables were problematic. For example, in places like the Northwest coast of North America, there are many fishing societies that live in densely packed coastal and/or riverine settlements with large, effectively empty adjacent territories (Ames, 2003; Binford, 2001). This fact makes the calculation of various spatial-demographic measures, above all population density, problematic. Second, in often being heavily dependent upon anadromous fish species—or what are sometimes characterized as “unearned resources” (Yesner, 1980)—there is little direct relationship between (terrestrial) environmental variables and subsistence dynamics. For example, along the Northwest coast of North America, the utilization of a common set of salmon fishing tactics ranges from Northern California through Western Alaska (Ames, 2003). Finally, such cases are also often characterized by high levels of social inequality that strongly affect mating/marriage systems and (ibid) which are likely not relevant to the terrestrial foraging and egalitarian human hunter-gatherer ancestors (Boehm, 1997, 2009).

In addressing this issue, I have eliminated hunter-gatherer cases in which more than 50% of calories are acquired from fishing activities. Clearly the elimination of cases is problematic and risks the appearance of “cherry picking.” To this end, I have repeated the analyses presented in this paper utilizing the full sample of hunter-gatherer societies, which is provided in Supplement 1. These results are discussed briefly below and in greater detail in the text of the supplement.

The data utilized in this analysis present other challenges from a statistical standpoint. First, none of the variables considered in this analysis follow a normal distribution and all follow a pattern characterized by low modal values and long “tails” in terms of the distribution of larger values. For this reason, conventional parametric statistical approaches, such as log transformation and linear regression, are not suitable for these data (see McCall, 2017 for a review). For this reason, the primary statistical method used in this paper is generalized linear modeling (GLM), which is an alternative nonparametric approach for quantifying the relationship between multiple variables (McCall & Villafranca, 2024). Another problem is that many of the variables considered in this analysis are strongly intercorrelated: environmental variables, subsistence variables, and social structural variables are all related to one another in complex ways and quite with relationships stemming from the phenomenon of variable latency. Multiple regression and path analysis are techniques that have been used to isolate and model the effects of individual variables within multivariate systems. Following from McCall and Marks (2024), this paper modifies the inputs for path analysis in replacing the traditional path coefficients derived from multiple linear regression standardized regression coefficients (Beta values) with rescaled exponentiated Beta values derived from multivariate GLM analysis.

## Data Analysis and Results

**Fig. 1:**
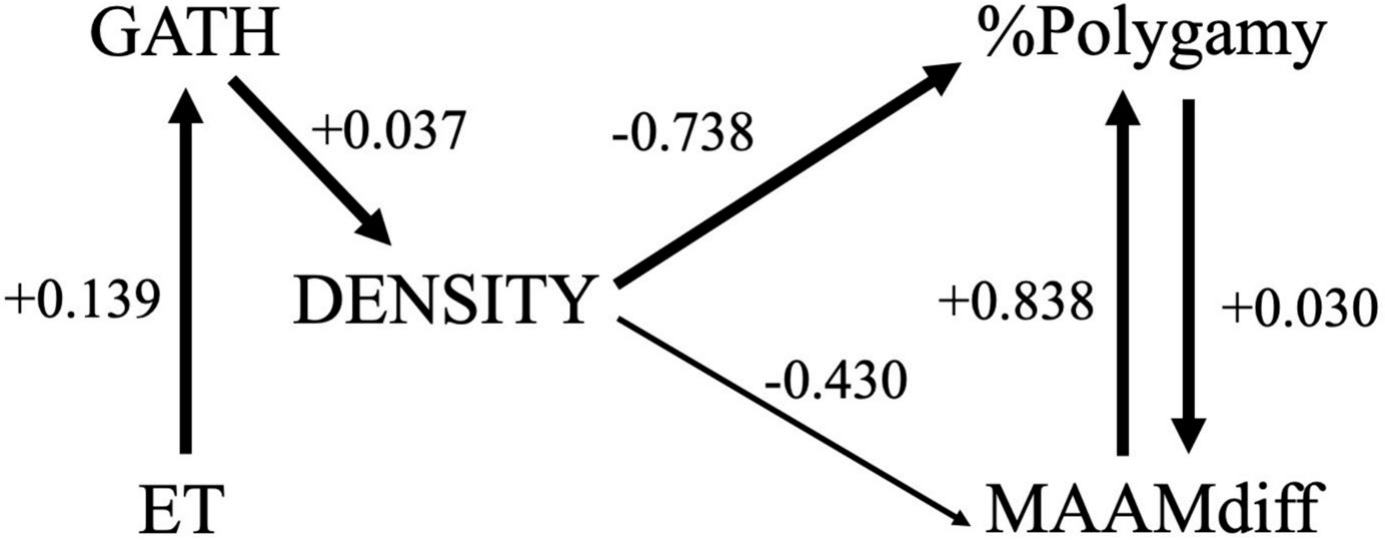
Path analysis for %Polygyny, MAAMdiff, DENSITY, GATH, and ET variables

**Fig. 2:**
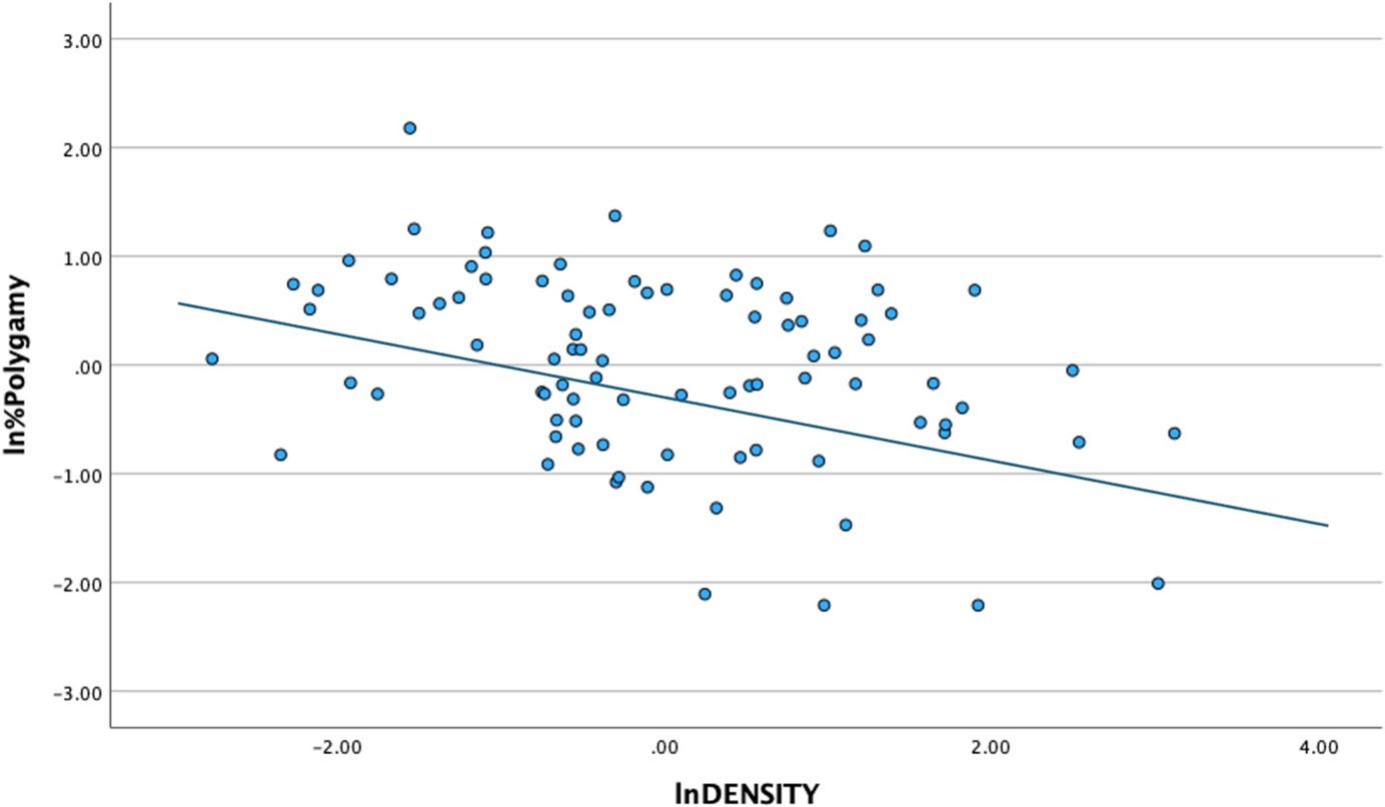
Partial multiple regression plot of the natural log (ln) of DENSITY and ln %Polygyny with other variables including ln MAAMdiff, ln GATH, and ln ET (*n* = 88; r.^2^ = 0.293; F = 8.589; *p* < 0.001)

Tables 1, 2,3, 4 and Fig. 1 show the results of the GLM and path analysis of the percentage of polygamous marriages (%Polygamy) and the mean difference in age at first marriage (MAAMdiff) based on population density (DENSITY), percentage of calories from gathering (GATH), and effective temperature (ET). The most striking result from this analysis is the extremely strong negative relationship between DENSITY and both %Polygamy and MAAMdiff. To summarize, the GLM models indicate a predicted decrease of approximately 43% and 74% for MAAMdiff and %Polygamy respectively for each increase of one person per km^2^. ET has a weakly positive direct effect on %Polygamy; GATH has a weak positive influence on DENSITY and ET has a weak positive relationship with GATH. Figure 2 shows a partial regression resulting from multiple regression analyses of the relationship between the natural log of DENSITY and the natural log of the %Polygamy. *This is done purely in order to present these statistical results graphically*. The major implication of this result is that population density is by far the strongest influence on hunter-gatherer mating systems, with other variables having weak and mostly indirect impacts.

**Table 1:** Results of GLM analysis of %Polygyny based on MAAMdiff, DENSITY, GATH, and ET variables

	B	Std. Error	Wald Chi-Square	df	Sig	Exp(B)
MAAMdiff	.609	.1631	13.932	1	<.001	1.838
DENSITY	−1.340	.3057	19.209	1	<.001	.262
GATH	-.003	.0046	.325	1	.569	.997
ET	.065	.0332	3.800	1	.051	1.067

*N* = 85; Likelihood ratio Chi-square = 39.482; df = 4; *p* < 0.001

**Table 2:** Results of GLM analysis of MAAMdiff based on %Polygyny, DENSITY, GATH, and ET variables

	B	Std. Error	Wald Chi-Square	df	Sig	Exp(B)
%polygny	.030	.0058	26.775	1	<.001	1.030
DENSITY	-.562	.2777	4.092	1	.043	.570
GATH	-.003	.0038	.625	1	.429	.997
ET	.023	.0284	.638	1	.425	1.023

*N* = 88; Likelihood ratio Chi-square = 42.721; df = 4; *p* < 0.001

**Table 3:** Results of GLM analysis of DENSITY based on GATH and ET variables

	B	Std. Error	Wald Chi-Square	df	Sig	Exp(B)
GATH	.036	.0048	57.304	1	<.001	1.037
ET	.009	.0254	.137	1	.711	1.009

*N* = 240; Likelihood ratio Chi-square = 67.873; df = 2; *p* < 0.001

**Table 4:** Results of GLM analysis of GATH based on ET variable

	B	Std. Error	Wald Chi-Square	df	Sig	Exp(B)
ET	.130	.0150	75.356	1	.000	1.139

*N* = 240; Likelihood ratio Chi-square = 76.650; df = 1; *p* < 0.001

**Fig. 3:**
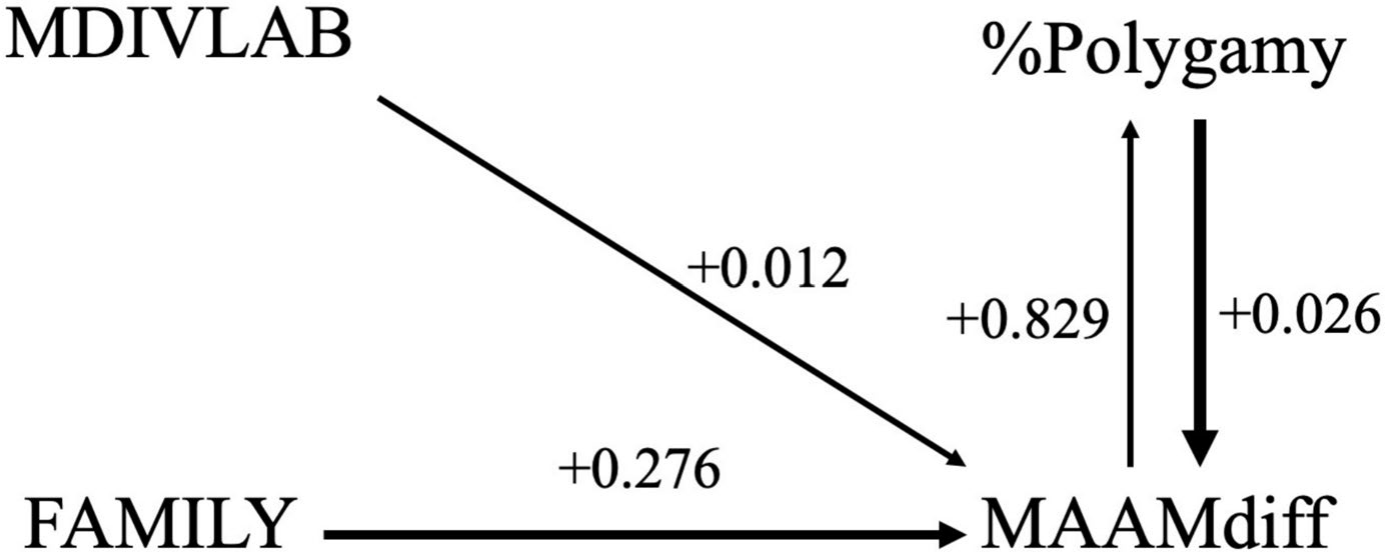
Path analysis for %Polygyny, MAAMdiff, FAMILY, and MDIVLAB variables

**Fig. 4:**
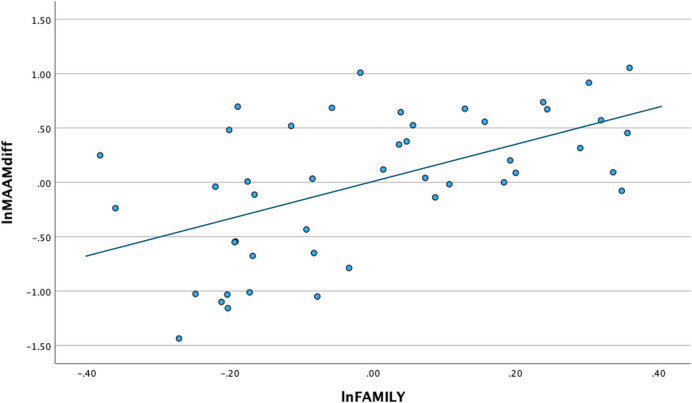
Partial multiple regression plot of the natural log of (ln) of FAMILY and ln MAAMdiff (*n* = 46; r^2^ = 0.457; F = 11.774; *p* < 0.001) including the %Polygamy and MDIVLAB variables

Next, Tables 5, 6 and Fig. 3 show results of the GLM and path analysis examining the relationships between %Polygamy, MAAMdiff, FAMILY, and MDIVLAB. Here, the strongest relationship is between family size and the mean difference in age at first marriage, in addition to a weaker relationship between male division of labor and the age at first marriage variable. Figure 4 shows a partial regression plot of the relationship between the natural log of FAMILY and the natural log of MAAMdiff, which is again *done purely for the sake of data illustration*. These results clearly demonstrate a major positive relationship between family size and delays in male age at first marriage, which in turn leads to higher frequencies of polygamous marriage. In other words, larger family sizes tend to correlate with longer delays in male age at first marriage and higher frequencies of polygamous marriages. Aspects of this patterning may seem somewhat paradoxical but it reduces to a rather important point: lower population densities foster conditions that lead to fewer but larger families associated with polygamous marriages, reflecting a higher degree of male-male competition in relation to the establishment of those marriages/families.

**Table 5:** Results of GLM analysis of %Polygyny based on MAAMdiff, FAMILY, and MDIVLAB variables

	B	Std. Error	Wald Chi-Square	df	Sig	Exp(B)
MAAMdiff	.604	.3016	4.005	1	.045	1.829
FAMILY	.139	.1382	1.013	1	.314	1.149
MDIVLAB	-.004	.0060	.458	1	.499	.996

*N* = 43; Likelihood ratio Chi-square = 16.084; df = 3; *p* = 0.001

**Table 6:** Results of GLM analysis of MAAMdiff based on %Polygyny, FAMILY, and MDIVLAB variables

	B	Std. Error	Wald Chi-Square	df	Sig	Exp(B)
%polygny	.026	.0073	12.523	1	<.001	1.026
FAMILY	.244	.0928	6.902	1	.009	1.276
MDIVLAB	.012	.0046	6.776	1	.009	1.012

*N* = 43; Likelihood ratio Chi-square = 35.284; df = 3; *p* < 0.001

The analyses reported in Supplement 1 utilizing the full sample of Binford’s (2001) hunter-gatherer data shows the same relationships, as well as similar values in terms of statistical significance and strength of association. The major difference has to with the impact of the environmental variables, particularly ET, on %Polygamy and MAAMdiff. The reason for this has to do largely with the inclusion of higher-latitude coastal cases in which levels of polygamy are low. Arguably, this pattern may have to do with limitations posed by male contributions to subsistence effort at the family unit level in terms of the role hunting and fishing and the lack of plant food gathering opportunities—a possibility I will return to below in my discussion of the “dependency ratio” explanation of hunter-gatherer polygamy (Binford, 2001: 221; Frost, 2008). In my view, it is also largely the result of problems with the calculation of population densities in higher-latitude coastal contexts, where large/densely populated villages are often situated in what are vast effectively empty territories. In any case, these analyses also show a strong relationship between population density hunter-gatherer mating structure.

## The Relationship Between Population Density and Hominin OSRs

The question of why higher hunter-gatherer population densities should lead to more balanced OSRs is an important one. There are a number of traditional explanations of variability in the prevalence of polygamy and, as this study shows, many would seem to hold value. In this respect, there are two basic and overlapping categories of issues influencing the structure of mating systems: First, there is direct reproductive decision-making, including female mate choice and interference from competing males. Second, there are organizational constraints stemming from the conduct of foraging activities and the environmental conditions that are responsible for structuring those activities. Such constraints have mostly to do with the gendered division of labor in terms of foraging, which also affects the structure of domestic labor in the rearing of offspring (among other things).

The conventional evolutionary ecological perspective on the effect of population size/density on mating systems has to do with direct intrasexual competition among males and interference stemming from a greater abundance of potential rivals. It is noteworthy, however, that for many non-human mammal species (including closely related primates such as chimpanzees; Boesch et al., 2006; Mitani, 2009), large population densities seem to have the opposite effect in terms of male-male competition and aggression (see also Wrangham, 2019). As both Trivers (1972) and Emlen and Oring (1977) pointed out early on, densely packed populations tend to occur in relation to abundant food resources, which are capable of energetically funding costly intrasexual competition and supporting large populations of conspecific potential mates (see also Kvarnemo & Ahnesjo, 1996). In this respect, intrasexual competition and territorial conflict are highly overlapping phenomena. Clearly, modern human hunter-gatherers diverge from this common pattern and the reasons for this likely hold heretofore under-examined relevance for our understanding of early hominin evolutionary ecology.

Female mate choice is an idea that has been emphasized previously providing a theoretical explanation for putative hominin self-domestication (Cieri et al., 2014; Hare, 2017; Gleeson & Kushnick, 2018; Gleeson & Wilson, 2023). In overview, the female mate choice perspective suggests that levels of male aggression reduced as females began to increasingly favor more sociable/cooperative males with a greater tendency to provide parental investment. Gleeson and Kushnick (2018) provide interesting evidence from modern hunter-gatherer societies demonstrating that sexual dimorphism varies in relation to female mate choice capacity. Such a finding suggests that polygamy can not only influence behavioral patterns and cultural mechanisms having to do with male aggression, but it also influences basic biological features related to size and strength. Though important questions remain (see Wrangham, 2019), the implications of these findings for the hominin self-domestication hypothesis are clear.

One idea about the relationships between population density, female mate choice, and OSRs has to do with issues of signaling and potential interference from intrasexual competitors (see Clutton-Brock, 2007). The root of this idea has to do with the importance of reproductive signaling in the context of intrasexual competition, including phenomena as diverse as physical ornamentation, handicap signaling, gift-giving, and intrasexual aggression (Bradbury & Vehrencamp, 1998; Johnstone, 1997; Smith & Harper, 2003; Zahavi, 1975). Among humans, though direct violent male-male competition for mates is rare, aggressive behavior and risk-taking as potential reproductive signals are much more common. Wilson and Daly (1985) saw this as the root of their “young male syndrome,” and Marlowe (2003) has shown that, among modern human hunter-gatherers, levels of violent behavior correlate strongly with the frequency of polygamous marriages. In this perspective, while young (unmarried) males may occasionally fight over potential mates directly, the overwhelming majority of aggressive behavior is done in the context of signaling to potential female mates.

As population density increases, so too does the signaling “noise” from competing males. When signals are effectively drowned out by noise from larger numbers of competing males, the incentive for signaling behavior is reduced. In short, as the number (or density) of competing males increases, the benefits of costly signaling decrease in terms of the likelihood that one would “stand out from the crowd” in attracting potential mates, while the costs of aggressive signaling in terms of potential injury, death, or embarrassing defeat remain. In such a context, it may also be the case that the valence of the reproductive signal itself is a problem: in a population packed with violent young males, displays involving the capacity for cooperation, parental investment, and social support—all key features of the female mate choice hypothesis (Cieri et al., 2014; Gleeson & Kushnick, 2018)—may be even more advantageous. In any case, they are certainly less physically risky and costly.

I would add a final consideration having to do with the relationship between hunter-gatherer mate selection dynamics and population density: sex ratios and stochasticity (Meggitt, 1965; Birdsell, 1968, 1973; Krupnik, 1985; Kramer et al., 2017). In his seminal paper in the *Man the Hunter* volume, Birdsell (1968) long ago pointed out that small hunter-gatherer groups are prone to random imbalances in sex ratios (since births and deaths are basically the flip of a coin). This is a major reason, he argued, that hunter-gatherers tend to maintain the “magic number” band and dialectical tribe sizes of 25 and 500 respectively. More recently, Kramer and colleagues (2017) show that, among the Pumé of Venezuela, major life decisions, especially the transfer of individuals from one foraging group to another, are strongly influenced by imbalances in adult sex ratios (ASRs), which are essentially the result of random life events.

There are also situations in which random fluctuations in group-level sex ratios can foster higher levels of polygamy given differences in male vs. female reproductive strategies (Kramer et al., 2017). Under conditions in which the availability of male mates becomes constrained, females may respond by joining existing marriages given the reproductive costs of delaying marriage. The reverse is not necessarily true: males almost never enter polyandrous marriages and are less time-constrained in waiting for effective ASRs to even out. Kramer and colleagues (2017) show that, among the Pumé—a low-latitude foraging society with relatively high population densities—potential marriage partners deal with stochastic variations in ASRs through mobility and flexibility in post-marital residence patterns.

In contrast, in Central Australia (Meggitt, 1965; Rose, 1969; Gould, 1969; Goodale, 1971; Marlowe, 2003; Marlowe & Berbeque, 2012), where forager population densities are very low and foraging groups are much more widely dispersed, mobility and post-marital residence options are not as easily available as ways of coping with imbalanced ASRs at the group level; or, at a minimum, these issues must be addressed with much greater lead time and longer-term planning. Here, male mate scarcity is often dealt with via the arrangement of polygamous marriages, which may in many cases occur at birth. In contrast, increased female mate scarcity simply results in longer delays in male first marriage, since such delays are less costly to males than they would be to females. This has the effect of intensifying male-male competition for mates (Marlowe, 2003; Marlowe & Berbesque, 2012) and clearly contributes to what Rose (1960, 1968) calls the “polygyny-gerontocracy” marriage politics common to forager societies in Central and Northern Australia. In this kind of situation, smaller population sizes and more isolated foraging groups make ASRs more prone to stochastic variation while also constraining mechanisms for coping with resulting problems. The result is the systematic delaying of male marriages and the utilization of polygamous marriage as a strategy for evening out stochastic variations among group-level ASRs. Large populations reduce stochastic ASR imbalances and provide other adaptive mechanisms involving transferal between foraging groups, as is described by Kramer and colleagues (2017).

The final perspective on the sources of polygamy among human hunter-gatherer societies is economic rather than reproductive. In this respect, Binford (2001) and Marlowe (2003) argue that a key determinate in the level of polygamy in forager societies has to do with the proportion of food supplied by males relative to that supplied by females, or what Binford (2001: 229) refers to as the “dependency ratio” (see also Frost, 2008). Binford and Marlowe contend that, in societies where males are the primary contributors to subsistence, polygamy tends not to occur by virtue of limitations on food availability. In other words, family size is limited by the amount of food that an individual male can produce. In contrast, in situations where females make relatively greater contributions of food resources, there is no limitation on the number of female mates within a family unit; and, in fact, family units may benefit from having more female foragers.

In its classic form, the male dependency hypothesis would not seem to have strong support from the data used in this analysis. There are no statistically significant influences on %Polygamy from either the GATH or MDIVLAB variables (Tables 1, 2, 3, 4, 5 and 6; Figs. 2 and 4).^1^ Male division of labor has a very weak but statistically significant relationship with difference in age at first marriage. Others who have found statistically significant relationships between foraging variables and polygamy/age at first marriage (Binford, 2001; Frost, 2008; Marlowe, 2003) did so using bivariate linear regression and it is well-known that subsistence variables are highly intercorrelated with male division of labor. The multivariate GLM methods used in this study suggest that foraging variables have a significant *indirect effect* on the polygamy/age at first marriage variables in being key correlates of population density.

With that said, I do continue to believe that subsistence limitations do play an important role in structuring hunter-gatherer mating systems. For example, there is an overwhelming pattern of monogamy and even sporadic polyandry among high-latitude foraging societies in North America and Eurasia. As Frost (2008) argues, female-biased OSRs result from a combination of near-exclusive reliance on male hunting and fishing activities in combination with high rates of male mortality/morbidity derived from exposure to risk of death/injury and wear-and-tear of long-distance foraging trips in extreme environmental conditions (see also Martin, 1974; Krupnik, 1985; Kelly, 1995; Binford, 2001; Starkweather & HAmes, 2012; Ziker et al., 2016).^2^ Though population densities are low in these cases by virtue of limited environmental productivity, the frequency of polygamous marriages is strongly constrained by dependency and limitations on male hunting/fishing success. This issue is discussed further in Supplement 1.

In thinking about the evolutionary past, all indications point to a pattern of food abundance based on the availability of high-ranked foraging resources using simple technologies and limited processing over the vast majority of the Pleistocene (Foley, 2001; Stiner & Munro, 2002; McCall, 2014; Holliday, 2023). Though the archaeological evidence along these lines is heavily tilted toward hunting, it is very likely that this is largely an issue of preservation and taphonomy—low-latitude Pleistocene hominin foragers likely accessed high-ranked plant food resources (and other subsistence opportunities), as do modern hunter-gatherers in the same environmental regions to this day. Hominin body size apparently reached its zenith during the Middle Pleistocene (and brain size followed suit shortly after; Churchill et al., 2012; Klein, 2009; McCall, 2014; Rightmire, 2004; Ruff et al., 2018). Clearly, Middle and early Upper Pleistocene hominins were burning large amounts of calories and acquiring food in sufficient quantities to leave body size relatively unconstrained—in ways that later Upper Pleistocene and Holocene human populations apparently were not. It seems very likely that there was sufficiently abundant food resources necessary for supporting imbalanced OSRs and equally unlikely that any sex/gender-based system of subsistence labor division would have constrained potential polygamy.

Given the combination of apparent food resource abundance and low population densities among Pleistocene early hominins, there is every reason to believe that our early hominin ancestors exhibited persistently and strongly imbalanced OSRs characterized by polygamous mating systems, delayed male ages at first marriage/mating, and therefore high levels of intrasexual competition characterized by aggressive conflict, risk-taking behavior, etc., concentrated among younger males. Such low population densities and imbalanced OSRs may well have been outside of the range of modern variability and the endurance of such conditions over the course of hundreds of thousands of years may have constituted a divergent selective context responsible for affecting both patterns of social behavior and (via the self-domestication hypothesis) anatomical characteristics including craniofacial robusticity.

## Population Density, Self-Domestication, and the Paleontological/Archaeological Record

Testing the role of population density as a potential cause of hominin self-domestication requires the fine-grained correlation of diachronic patterning across the domains of hominin demographics and robusticity. There is, of course, a general intuitive belief that hominin population sizes increased over time across the Pleistocene, and that belief has been used to support many arguments put forward to explain key prehistoric phenomena (see French, 2016 for a review). The relationship between population density and OSRs described above also provides potentially testable propositions having to do with the hominin self-domestication hypothesis. Unfortunately, much of what we believe about Pleistocene hominin demographic patterning is rather speculative—for example, Hassan’s (1981) influential Paleolithic population estimates. Direct evidence, such as site counts, chronometric date frequencies, and deposit accumulation sizes are themselves problematic and hindered by issues of archaeological visibility and dating difficulties, particularly for contexts beyond the radiocarbon limit (Bocquet-Appel & Demars, 2000). More remote periods of the Pleistocene present even more difficulties in terms of scale and resolution in identifying periods of hominin population growth (or decline) and correlating those with patterns of variation in craniofacial robusticity.

Yet, for the Upper Pleistocene and Holocene, some creative attempts have made progress in addressing this issue based on hominin populational impacts on ecodynamic proxies. For example, Stiner and colleagues (1999, 2000) utilize data concerning the exploitation of small terrestrial fauna in Europe and the Near East to infer a series of population surges during the Upper Paleolithic with major implications for modern human origins and the “broad spectrum” foraging revolution. Klein and Steele (2013) similarly focus on shellfish and tortoise sizes as proxies for human population densities, demonstrating a major demographic increase in the transition from the Middle to Later Stone Age in the Cape region of South Africa (see also Steele & Klein, 2005). Similarly, at the global scale, Kirch (2005) reviews the massive ecodynamic impacts resulting from profound increases in Holocene human populations in the context of emerging agricultural systems and other features of “post-Pleistocene adaptation” (Binford, 1968). At present, I believe that few would doubt that there were major inflections in human population growth at various points during the late Upper Pleistocene and Holocene.

Another set of problems for the hominin self-domestication hypothesis involves the issue of characterizing craniofacial robusticity, as well as potentially related features, such as body size/dimorphism and brain size. Craniofacial robusticity is viewed as a suite of numerous putatively related features of the head and face, including thick/prominent brow ridges, occipital tori, and cranial vault, as well as *many* others (see, for example, Lahr & Wright, 1996). Body size/dimorphism is the most straightforwardly observable feature among living human groups having to do with intrasexual competition and physical aggression (Gleeson & Kushnik, 2018)—though the relationship between body size/dimorphism and robusticity remains unclear. Finally, brain size is another biological feature that reduces slightly in perhaps unexpected ways among late Upper Pleistocene human populations and that has been linked with the domestication syndrome (Wilkins et al., 2014; Hare, 2017; Sánchez-Villagra & Schaik, 2019; Gleeson & Wilson, 2023).

In my view, the strongest prehistoric evidence for the hominin self-domestication hypothesis begins among modern humans in the late Upper Pleistocene and Holocene, when both appropriate skeletal remains and archaeological evidence germane to demographic inference are at their most abundant. Here, there would seem to be clear correlations between the anatomical shifts at stake in the hominin self-domestication hypothesis and major human population increases. As Lahr and Wright (1996) put it, “The ancestral condition for early modern humans is one of large [cranial] size (in both breadth and length) and robusticity, and the later differentiation of populations must have occurred partly through reduction along these parameters.” Body size and brain size also reduce significantly among late Upper Pleistocene and Holocene human populations (Bruner & Gleeson, 2019; DeSilva et al., 2021; Henneberg, 1988; Leach, 2003; McCall, 2014; Ruff, 2002). Thus, the co-occurrence of such anatomical features of hominin self-domestication and late Upper Pleistocene-to-Holocene human demographic expansions is striking and frames this context for further research.

With that said, the population density variant of the hominin self-domestication hypothesis is clearly relevant to anatomical and potentially cultural patterning evident among archaic hominin populations. For example, Neanderthals and modern humans both putatively descended from a Middle Pleistocene hominin ancestor and have a great deal in common, both biologically—for example, equivalent brain sizes (Ponce de León et al., 2016)—and culturally—for example, the design of hunting tools and weapons (Villa & Soriano, 2010). Yet, Neanderthals and modern humans differ anatomically in major ways and craniofacial robusticity constitutes one of the most significant sets of differences in this regard. Though biomechanical/environmental explanations of such differences in craniofacial robusticity have enjoyed popularity (e.g. Smith, 1983), it is also easy to conceive of enduring demographic differences between densely packed core populations of early modern humans in the low latitudes of sub-Saharan Africa and the sparser higher-latitude Neanderthal populations of Europe and Western Asia. In such a way, differences in population density between Neanderthals and early modern humans may have fostered differently structured mating systems and OSR imbalances, resulting in greater expression of various features of self-domestication among early modern humans.

It may be the case that population density can also provide a framework for applying the hominin self-domestication model to Lower and Middle Pleistocene hominin populations, as has been suggested by authors such as Wrangham (2019) and Boehm (1997, 2009) via the reverse-dominance hierarchy idea. Again, a combination of low population densities and highly effective foraging activities (see McCall, 2014; Holliday, 2023) may have set the stage for imbalanced OSRs among Lower and Middle Pleistocene hominin populations. It is in this context that the striking pattern of hominin encephalization occurs and in which hominins achieve both their peak body size and most extreme robusticity (Klein, 2009; Ruff, 2002). From there, it is plausible that population expansions, such as one putatively involved in the origins of *Homo sapiens* from a Middle Pleistocene ancestor such as *Homo heidelbergensis*, played a role in the biological changes associated with self-domestication. In this sense, hominin self-domestication, in resulting from shifting OSRs caused by changes in population density, need not be views as a kind of synchronic threshold but rather an ongoing set of evolutionary processes organizationally relating to a range of salient biological and cultural features.

## Conclusion

This paper has offered a theoretical scenario linking hominin population density, mating systems, aggressive intrasexual competition, and evolutionary processes related to self-domestication. In a previous criticism of population density as a theoretical explanation of self-domestication, Wrangham (2019: 7) states the following: “The population density hypothesis… fails to detail any process responsible for selection directly against alpha-male-style behavior.” That may be true from a certain point of view; however, this paper has shown that, among modern hunter-gatherers, population density plays a key role in structuring fundamental aspects of mating systems and patterns of intrasexual competition between males. This clearly suggests that population density influences selective conditions having to do with dynamics of male intrasexual competition, potentially providing a crucial basis for a shared disruption to reproductive systems that could be responsible for the emergence of features of self-domestication (Gleeson & Wilson, 2023).

This theoretical scenario is appealing because it provides so-far elusive connections between the most visible biological characteristics of early hominins in terms of robusticity, the structure of hominin mating systems and their consequences for aggressive behavior, and the broader organizational features of Paleolithic foraging systems (Leach, 2003). The anterior dental loading hypothesis and the teeth-for-tools ideas were successful in the past because they provided direct adaptive explanations for otherwise puzzling features of hominin robusticity. They ultimately remained unsatisfying (at least in my view) for the best of all possible scientific reasons: in the end, as evidence continued to accumulate, that evidence increasingly contradicted the theoretical scenario. The self-domestication hypothesis provides an alternative that clearly has compelling aspects in terms of explaining the biological basis for robusticity, however, many of the causal mechanisms invoked by this hypothesis have remained speculative and generally far less compelling (see Sánchez-Villagra & Schaik, 2019). As this paper has shown, the behavioral ecology of modern human hunter-gatherers provides one approach for improving such theoretical explanations (see also O'Connell, 1995), although more complex synthetic approaches to the prehistoric evidence are also needed.

## Supplementary Information

Below is the link to the electronic supplementary material.

Supplementary file1 (DOCX 302 KB)

## Data Availability

The data used in this paper will be made available upon request to the author.

## Footnotes

1 When the full Binford (2001) hunter-gatherer sample is considered, there is a weakly statistically significant relationship between GATH and %Polygamy (Exp. Beta = 0.992, p = 0.030) and more strongly significant relationship between GATH and MAAMdiff (Exp. Beta = 0.990, p = 0.001). There is a weak but statistically negative significant relationship between MDIVLAB and %Polygamy (Exp. Beta = 0.990, p = 0.048), which is the opposite of the dependency ratio prediction. There are stronger positive relationships between both MDIVLAB and FAMILY and MAAMdiff (respectively: Exp. Beta = 1.011, p = 0.001; Exp. Beta = 1.266; p = 0.001). See Supplement 1 for further discussion.

2 The results the analysis of the full Binford (2001) hunter-gatherer sample would seem to support this hypothesis, although there may be other issues at play in structuring this relationship. See Supplement 1 for further discussion.
