# Supplementary material for: Hominin Population Structure, Mating Systems, and Intrasexual Competition: Implications for Craniofacial Robusticity and the Self-Domestication Hypothesis
Source: Hum Nat. 2025 Jul 25;36(2):307–35. doi: 10.1007/s12110-025-09498-6 (PMC12417227; doi:10.1007/s12110-025-09498-6)
Supplement: Supplementary file 1 — Supplementary file1 (DOCX 302 KB) [file 12110_2025_9498_MOESM1_ESM.docx]

**Supplementary Material**

This supplement provides the identical analyses presented in the main paper, however, including the full sample of Binford’s (2001) hunter-gatherer data. The analyses presented here reach very similar results, particularly in relation to the effect of population density (DENSITY) on the operational sex ratio (OSR) variables (%Polygamy and MAAMdiff). DENSITY again has a strong negative relationship with %Polygamy, with an exponentiated beta value of 0.491, meaning that for every increase of one person per km^2^, %Polygamy is predicted to decrease by ~51%. DENSITY has a somewhat weaker but statistically significant relationship with MAAMdiff, with an exponentiated beta value of 0.670.

The main difference between the two analyses is the effect of effective temperature (ET) on %Polygamy. In the analysis of the non-coastal sample presented in the paper, no statistically significant relationship exists between ET and either of the OSR variables. For the full sample, ET has no significant relationship with MAAMdiff but it does have a fairly strong relationship with %Polygamy (exp. Beta = 0.903). On the one hand, this may have to do with a real and potentially important relationship with gendered division of labor and the male dependency ratio hypothesis. For example, Arctic foragers tend to focus nearly exclusively on hunting and fishing, which are often done in risky and dangerous context and exclusively by men. Polygamy would indeed seem to be limited by male contributions to subsistence at the family level and, in fact, this is one of the rare contexts in which polyandry occurs. On the other hand, the problematic group of coastal foragers, particularly on the Northwest Coast of North America, are also characterized by low levels of polygamy and low ET values, *but also* relatively low DENSITY values. In that sense, the problematic population density values may be at least partly responsible for a misleading relationship between ET and %Polygamy.

Finally, it is important to notice that the effects of DENSITY and ET on %Polygamy are contradictory in important ways. That is, there is (1) an obvious direct relationship between ET and DENSITY, since warmer environments support higher plant productivity, greater food availability, and larger human populations (see Binford 2001); but (2) ET has a strong inverse relationship with %Polygamy, as does DENSITY. In my view, this is very greatly the result of the problematic population density values for the fishing-heavy coastal hunter-gatherer groups, particularly along the Northwest Coast, and this latter relationship disappears when the coastal societies are removed from the sample.

Figure S1. ﻿Path analysis for %Polygyny, MAAMdiff, DENSITY, GATH, and ET variables with the entire Binford (2001) dataset included.


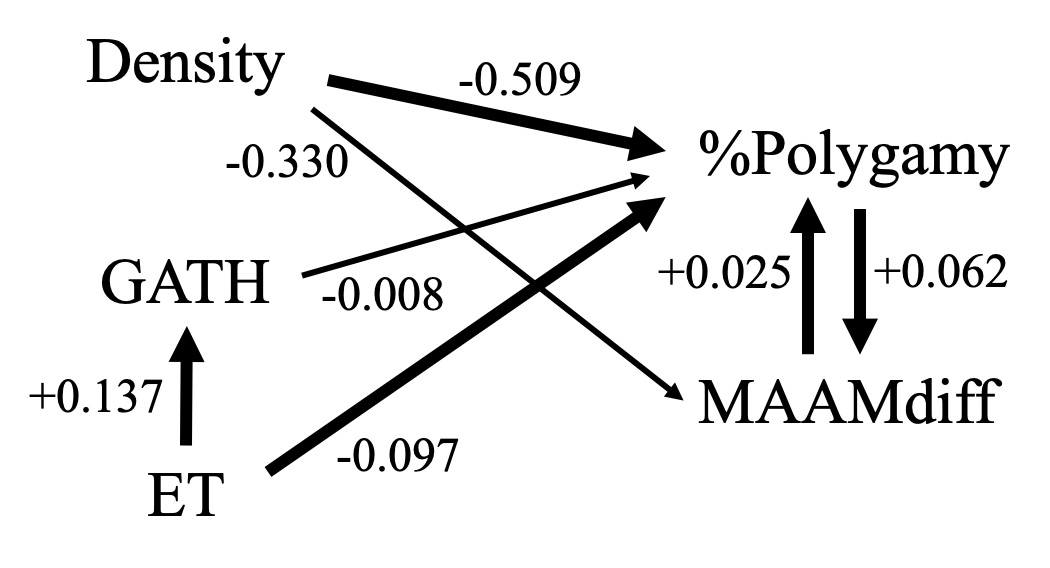


Figure S2. ﻿Path analysis for %Polygyny, MAAMdiff, FAMILY, and MDIVLAB variables with the entire Binford (2001) dataset included.


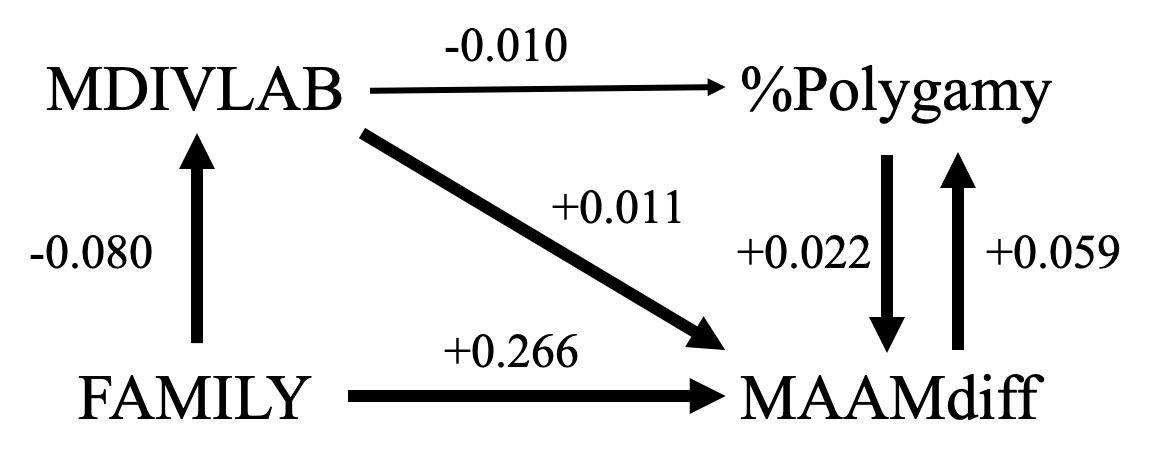


Table S1. ﻿Results of GLM analysis of %Polygyny based on MAAMdiff, DENSITY, GATH, and ET variables. N = 122; Likelihood ratio Chi-square = 61.576; df = 3; p < 0.001.

| Parameter | B | Std. Error | Hypothesis Test | | | Exp(B) |
| --- | --- | --- | --- | --- | --- | --- |
|  |  |  | Wald Chi-Square | df | Sig. |  |
| (Intercept) | 1.201 | .2634 | 20.803 | 1 | <.001 | 3.324 |
| MAAMdiff | .060 | .0119 | 25.545 | 1 | <.001 | 1.062 |
| DENSITY | -.711 | .2581 | 7.589 | 1 | .006 | .491 |
| GATH | -.008 | .0036 | 4.722 | 1 | .030 | .992 |
| ET | .092 | .0238 | 15.107 | 1 | <.001 | 1.097 |
| (Scale) | 1.925^a^ | .2103 |  |  |  |  |

Table S2. ﻿Results of GLM analysis of %Polygyny based on MAAMdiff, DENSITY, GATH, and ET variables. N = 122; Likelihood ratio Chi-square = 56.149; df = 3; p < 0.001.

| Parameter | B | Std. Error | Hypothesis Test | | | Exp(B) |
| --- | --- | --- | --- | --- | --- | --- |
|  |  |  | Wald Chi-Square | df | Sig. |  |
| (Intercept) | 1.434 | .2196 | 42.648 | 1 | <.001 | 4.196 |
| %polygamy | .025 | .0040 | 38.555 | 1 | <.001 | 1.025 |
| DENSITY | -.400 | .1946 | 4.221 | 1 | .040 | .670 |
| GATH | -.004 | .0029 | 1.791 | 1 | .181 | .996 |
| ET | .015 | .0210 | .529 | 1 | .467 | 1.015 |
| (Scale) | .835^a^ | .0987 |  |  |  |  |

Table S3. ﻿Results of GLM analysis of %Polygyny based on MAAMdiff, DENSITY, GATH, and ET variables. N = 339; Likelihood ratio Chi-square = 11.193; df = 2; p = 0.004.

| Parameter | B | Std. Error | Hypothesis Test | | | Exp(B) |
| --- | --- | --- | --- | --- | --- | --- |
|  |  |  | Wald Chi-Square | df | Sig. |  |
| (Intercept) | -1.971 | .2206 | 79.799 | 1 | <.001 | .139 |
| GATH | .003 | .0027 | 1.383 | 1 | .240 | 1.003 |
| ET | .031 | .0173 | 3.191 | 1 | .074 | 1.031 |
| (Scale) | .461^a^ | .0231 |  |  |  |  |

Table S4. ﻿Results of GLM analysis of %Polygyny based on MAAMdiff, DENSITY, GATH, and ET variables. N = 339; Likelihood ratio Chi-square = 163.554; df = 1; p < 0.001.

| Parameter | B | Std. Error | Hypothesis Test | | | Exp(B) |
| --- | --- | --- | --- | --- | --- | --- |
|  |  |  | Wald Chi-Square | df | Sig. |  |
| (Intercept) | 1.537 | .1503 | 104.581 | 1 | <.001 | 4.649 |
| ET | .128 | .0093 | 191.003 | 1 | <.001 | 1.137 |
| (Scale) | 2.500^a^ | .1564 |  |  |  |  |

Table S5. Results of GLM analysis of %Polygyny based on MAAMdiff, DENSITY, GATH, and ET variables. N = 61; Likelihood ratio Chi-square = 33.735; df = 1; p < 0.001.

| Parameter | B | Std. Error | Hypothesis Test | | | Exp(B) |
| --- | --- | --- | --- | --- | --- | --- |
|  |  |  | Wald Chi-Square | df | Sig. |  |
| (Intercept) | 1.936 | .6446 | 9.015 | 1 | .003 | 6.928 |
| MAAMdiff | .057 | .0208 | 7.592 | 1 | .006 | 1.059 |
| FAMILY | .164 | .1159 | 2.001 | 1 | .157 | 1.178 |
| MDIVLAB | -.010 | .0051 | 3.903 | 1 | .048 | .990 |
| (Scale) | 2.048^a^ | .3113 |  |  |  |  |

Table S6. Results of GLM analysis of %Polygyny based on MAAMdiff, DENSITY, GATH, and ET variables. N = 61; Likelihood ratio Chi-square = 46.571; df = 1; p < 0.001.

| Parameter | B | Std. Error | Hypothesis Test | | | Exp(B) |
| --- | --- | --- | --- | --- | --- | --- |
|  |  |  | Wald Chi-Square | df | Sig. |  |
| (Intercept) | -.152 | .4014 | .144 | 1 | .704 | .859 |
| %polygny | .022 | .0055 | 15.573 | 1 | <.001 | 1.022 |
| FAMILY | .236 | .0708 | 11.122 | 1 | <.001 | 1.266 |
| MDIVLAB | .011 | .0033 | 11.059 | 1 | <.001 | 1.011 |
| (Scale) | .600^a^ | .1031 |  |  |  |  |

Table S7. Results of GLM analysis of %Polygyny based on MAAMdiff, DENSITY, GATH, and ET variables. N = 114; Likelihood ratio Chi-square = 8.945; df = 1; p = 0.003.

| Parameter | B | Std. Error | Hypothesis Test | | | Exp(B) |
| --- | --- | --- | --- | --- | --- | --- |
|  |  |  | Wald Chi-Square | df | Sig. |  |
| (Intercept) | 4.523 | .1271 | 1266.988 | 1 | <.001 | 92.066 |
| FAMILY | -.083 | .0272 | 9.282 | 1 | .002 | .920 |
| (Scale) | .747^a^ | .0971 |  |  |  |  |
